# Phase Coexistence in Thermoresponsive PNIPAM Hydrogels Triggered by Mechanical Forces

**DOI:** 10.1021/acs.macromol.5c03088

**Published:** 2026-02-05

**Authors:** Noy Cohen

**Affiliations:** Department of Materials Science and Engineering, 26747 Technion - Israel Institute of Technology, Haifa 3200003, Israel

## Abstract

Poly­(*N*-isopropylacrylamide) (PNIPAM) is a temperature-responsive polymer that undergoes large volumetric deformations through a transition from a swollen to a collapsed state at a volume phase transition temperature (VPTT). Locally, these deformations stem from the coil-to-globule transition of individual chains. In this contribution, I revisit the study of SuzukiA., ; IshiiT., [J. Chem. Phys. 1999, 110, 2289–2296 ], which demonstrated that a PNIPAM rod can exhibit phase coexistence (i.e., comprise swollen and collapsed domains simultaneously) near the VPTT when subjected to mechanical constraints. Specifically, that paper showed that (1) collapsed domains gradually form in a fixed swollen rod with time and (2) swollen domains can nucleate in a collapsed rod under uniaxial extension. These behaviors originate from the local thermo-mechanical response of the chains, which transition between states in response to the applied mechanical loading. Here, I develop a statistical-mechanics based framework that captures the behavior of individual chains below and above the VPTT and propose a probabilistic model based on the local chain response that sheds light on the underlying mechanisms governing phase nucleation and growth. The model is validated through comparison with experimental data. The findings from this work suggest that in addition to the classical approaches, in which the VPTT is programmed through chemical composition and network topology, the transition can be tuned by mechanical constraints. Furthermore, the proposed framework offers a pathway to actively tailor the VPTT through the exertion of mechanical forces, enabling improved control and performance of PNIPAM hydrogels in modern applications.

## Graphical abstract

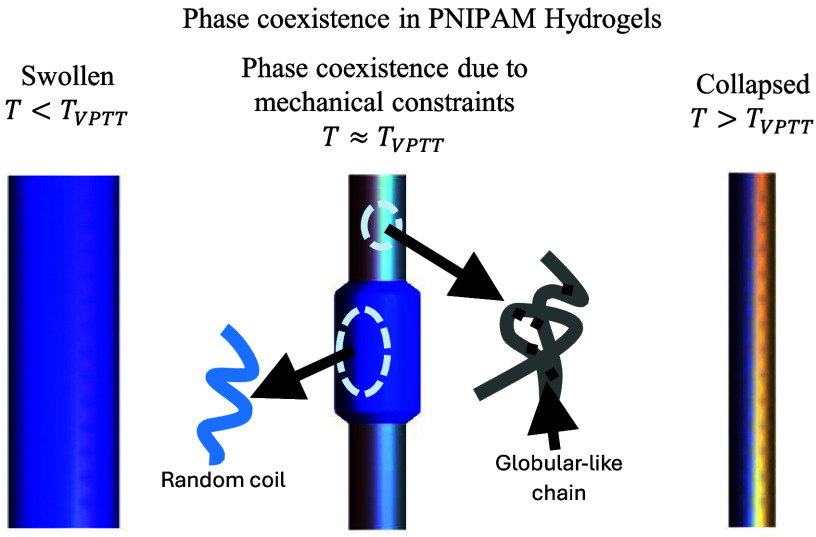

## 1 Introduction

Poly­(*N*-isopropylacrylamide) (PNIPAM) is a thermoresponsive macromolecule that undergoes a local reversible coil-to-globule transition on the chain level and a macroscopic swollen-to-collapsed volumetric deformation at a volume phase transition temperature (VPTT).^2–4^ Typically, the VPTT is *T*_VPTT_ ∼ 32–34 °C, with variations that depend on different factors such as composition,^5–7^ solvent type,^8,9^ and the application of external force.^1,6^ The volumetric deformations associated with the transition are significant, with the literature reporting over a 10 times increase in volume from the collapsed to the swollen state.^4,6,10–12^

The sharp thermally induced phase transition in PNIPAM hydrogels lends itself to many applications. For example, PNIPAM hydrogels have been proposed for tissue engineering^13–15^ and drug delivery systems,^16–18^ in which the temperature-dependent response is exploited for cell scaffolding and controlled release. In soft robotics, PNIPAM hydrogels are used in the design of shape-memory materials,^19^ smart switches,^20^ and deformable structures that exploit the temperature-dependent volume changes.^21,22^

Interest in PNIPAM hydrogels has steadily increased over the past decades. Beginning with the pioneering work of Hirokawa and Tanaka,^23^ who demonstrated the sharp volume phase transition in nonionic gels, many experiments and modeling attempts have been carried out. At the chain-level perspective, several studies focused on the local response before and after the VPTT.^12,24–27^ The bulk response of PNIPAM gel was investigated through continuum-based models,^10,12,25,28–31^ as well as different experimental set-ups.^1,6,11,32^

The behavior of PNIPAM hydrogels has been traditionally characterized by their response at states below and above the VPTT, with the transition captured via changes in the phenomenological dimensional interaction parameter χ, as proposed by Flory.^33,34^ Levin and Cohen^25^ established a fundamental framework to capture the network response by focusing on how microstructural quantities such as chain length and distribution evolve and govern the equilibrium response, and accordingly described the transition from collapsed to swollen and vice versa. However, a significant challenge remains in understanding the microstructural evolution of the network and the influence of mechanical forces around the transition region itself.

The insightful work of Suzuki and Ishii^1^ experimentally demonstrated that around the VPTT an applied mechanical loading can induce phase coexistence, i.e., the simultaneous and stable presence of collapsed and swollen phases, as shown in Figure 1. This contribution revisits that paper by building on the findings of Levin and Cohen^25^ and introducing a framework that delineates the underlying mechanisms governing phase coexistence by explicitly accounting for the stochasticity of conformational chain transitions and the influence of external forces. This addition is critical to capturing the nucleation and growth of new phases, as it provides a quantifiable description of the transition regime and enables one to tune the VPTT through mechanical forces.

**Figure 1:**
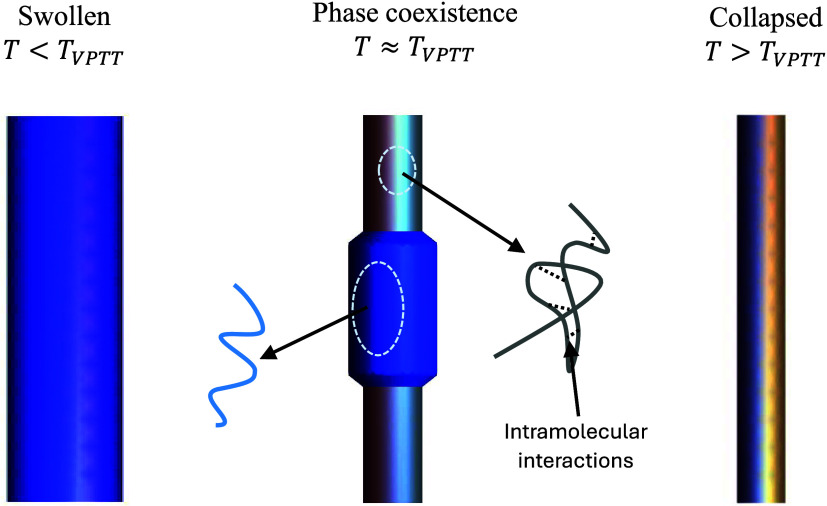
The swollen phase, phase coexistence, and the collapsed phase of a PNIPAM rod.

### 1.1 Phase Coexistence in PNIPAM Networks

We recall that a PNIPAM hydrogel that is subjected to a temperature *T* < *T*_VPTT_ is in a swollen state while at temperatures *T* > *T*_VPTT_ the network is in a collapsed configuration. Phase coexistence, or the existence of a swollen and a collapsed phase simultaneously, in PNIPAM rods was reported to occur in response to a mechanical force around the VPTT (i.e., *T* ≈ *T*_VPTT_) by Suzuki and Ishii.^1^ The phase coexistence is illustrated in Figure 1. To demonstrate this phenomenon, Suzuki and Ishii^1^ measured *T*_VPTT_ ≈ 33.5 °C and performed two types of experiments with different boundary conditions at *T* ≈ *T*_VPTT_ - (1) time-dependent phase coexistence in fixed PNIPAM rods and (2) stretch-induced phase coexistence.

In the first experiment, a swollen PNIPAM rod was fixed to a constant (relaxed) length at *T* = 30 °C and heated up to *T* = 33.5 °C. At the beginning of the experiment (*t* = 0), the rod was fully swollen. As time progressed, a collapsed phase began to develop in the swollen rod and slowly grow until a steady state was achieved. After 200 h, the rod exhibited a stable configuration with phase coexistence that lasted several days. In the second experiment, a collapsed PNIPAM rod was placed at a constant temperature *T* = 33.5 °C and uniaxially stretched. Under small stretches, the rod elongated while remaining in a collapsed configuration. Once a critical stretch was applied, a swollen domain developed in the rod with a size that further increased with stretch. The exertion of a sufficiently large force led to the complete transition of the rod into a single-phase swollen configuration. It is worth mentioning that in both experiments raising or decreasing the temperature by 0.1 °C led to the full transition of the rod from one state to another.

The origin of this phenomenon is in the mechanically induced local transition of chains from a globule-like to a coil conformation, depicted in Figure 2. To understand this, recall that chains in the collapsed (globule-like) state form intramolecular interactions that develop between spatially close hydrophilic side groups and act as physical cross-links.^35–37^ These interactions essentially cause the chain to fold and shield the hydrophobic groups from water by assembling hydrophobic clusters.^25,38,39^ As a consequence, collapsed chains are hydrophobic, have a shorter effective contour length, and limited mobility. The application of a sufficiently large local force on the chain can disrupt and break the intramolecular bonds, exposing the hydrophilic side groups.^25,26,35,37,40–42^ This results in two main consequences - the chain becomes hydrophilic and attracts water molecules and the contour length of the chain increases.

**Figure 2:**
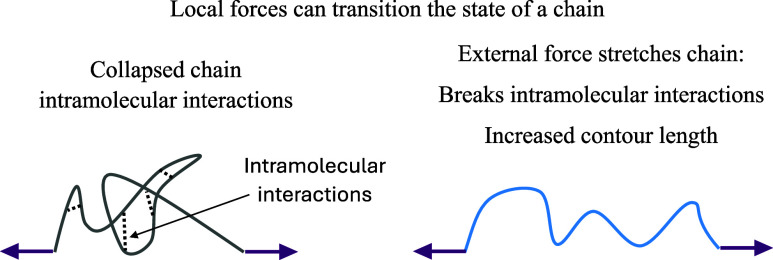
Stretching of a “collapsed” chain to induce an extended chain by breaking the intramolecular bonds at temperature *T* > *T*_VPTT_.

At the network level, the mechanically induced transition of a sufficient number of chains from one state to another marks the nucleation of a local transition. For example, stretching a collapsed rod around the VPTT leads to the local extension of chains, which transition from globule-like to coil. In turn, the exposure of the hydrophilic amide side groups increases attraction to water molecules and motivates water uptake, ultimately leading to the formation of a swollen domain in a collapsed PNIPAM rod. In the case of a swollen network that is fixed in length in an environment with a temperature around the VPTT, the thermal energy works toward contracting the rod. The thermal energy works toward transitioning the chains to a collapsed state, and over time collapsed domains form in the swollen bulk.

This work aims to provide a better understanding of the underlying mechanisms that enable phase coexistence in PNIPAM networks. In the following, I begin by developing a constitutive local model for the collapsed and the swollen chains and networks. Next, I phrase the conditions for the coexistence of phases and compare our model predictions to experimental findings.

## 2 Collapsed versus Swollen Phases in PNIPAM Networks

The work of Levin and Cohen^25^ modeled the mechanical response of PNIPAM networks and the temperature-induced swollen-to-collapsed transition by introducing explicit relations that capture the dependency of the effective contour length and the number of repeat units in a chain on temperature. The aim of the current contribution is to explain the origin of the coexistence of phases around the VPTT, and therefore I begin by developing constitutive relations for the chain and the network response in each of the phases separately. The chains are modeled as freely jointed with contour lengths and number of repeat units that vary in the coil and the globule-like states.

First, we recall the governing equations of polymer networks comprising freely jointed chains. The entropy of a freely jointed chain with *n* repeat units and a contour length *L* ^43–45^

1 $$S_{c}\left(\rho \right)=\mathrm{const}-k_{b}n\left(\rho \beta \left(\rho \right)+\ln\left(\frac{\beta \left(\rho \right)}{\sin h\left(\beta \left(\rho \right)\right)}\right)\right)$$

where *k*_*b*_ is the Boltzmann constant, ρ = *r*/*L* is the ratio between the end-to-end distance and the contour length of the chain, and the function β is determined from the Langevin function ρ = cot h β – 1/β, which can be approximated via β ≈ ρ­(3 – ρ^2^)/(1 – ρ^2^).^46^

The free energy associated with the chains can be written as ψ_*c*_ = – *TS*_*c*_, and accordingly the force required to maintain an end-to-end distance *r* is^44,45^

2 $$f_{c}\left(\rho ,n\right)=\frac{\partial \psi_{c}}{\partial r}=k_{b}T\frac{n}{L}\beta \left(\rho \right)$$

To model the macroscopic response, consider a network with *N*_0_ chains per unit dry volume subjected to a mechanical force at temperature *T*. As a result of a thermo-mechanical loading, the network experiences the macroscopic deformation gradient **F**. To integrate from the chain to the network level, consider a chain with an initial end-to-end vector **R** = *R* **R̂**, where R = *L*/√*n* is the initial end-to-end distance and **R̂** is the direction. Following common practice,^44,45^ I assume that the chains experience the macroscopic deformation gradient such that the deformed end-to-end vector is **r** = **FR** with a length *r* and the interactions between the chains are negligible. Accordingly, the total entropy of a referential volume element *dV*_0_ is

3 $$S\left(F,R\right)=\frac{1}{dV_{0}}\sum_{i}S_{c}^{\left(i\right)}=N_{0}\left\langle S_{c}\right\rangle$$

where the summation is carried over all chains and ⟨*S*_*c*_⟩ is the average entropy. Henceforth, the average of a quantity • is denoted by ⟨•⟩ = (∑_*i*_ *S*_*c*_^(*i*)^)/*N*_0_ d*V*_0_. Subsequently, the total energy free energy-density of the network due to the deformation of the chains is

4 $$\psi_{n}\left(F,R,T\right)=-\mathrm{TS}$$

In the following, we employ the above formulation to develop the energy-density functions for the swollen (*T* < *T*_VPTT_) and the collapsed (*T* > *T*_VPTT_) states in PNIPAM networks.

### 2.1 The Swollen State (*T* < *T*_VPTT_)

Consider a dry PNIPAM network under a temperature *T* < *T*_VPTT_ comprising *N*_0_ chains per unit dry volume. In the undeformed dry state, the material points are denoted by **x**_*s*_. The network is placed in an aqueous bath and allowed to swell under mechanical forces. Due to the water uptake and the application of a mechanical load, the hydrogel deforms such that in the current configuration its material points are denoted by **y**_*s*_. The deformation gradient is defined as **F**_*s*_= ∂**y**_*s*_/∂**x**_*s*_ = *J*_*s*_^1/3^ **F̃**_*s*_, where det **F**_*s*_ = *J*_*s*_ is the volumetric deformation due to swelling and det **F̃**_*s*_ = 1 is the deformation due to the isochoric distortion of the network. The density of water molecules per unit dry volume in the swollen gel is denoted *m.*

From a microscopic viewpoint, the average chain comprises *n*_*s*_ repeat units with a contour length *L*_*s*_, and accordingly the average end-to-end distance is *R*_*s*_ = *L*_*s*_/*n*_*s*_. The force on a chain is given by *f*_*s*_ = *f*_*c*_ (ρ_*m*_, *n*_*s*_), where *f*_*c*_ is given in eq 2 and ρ_*m*_ = *r*/*L*_*s*_.

The total energy-density of the swollen network can be written as^47^

5 $$\psi_{s}\left(F_{s},T\right)=\psi_{n}\left(F_{s},R_{s},T\right)+\psi_{m}\left(J_{s},T\right)-p_{s}\left(J_{s}-\frac{1}{c_{p}}\right)$$

where the first term is given in eq 4 with *n* = *n*_*s*_, the second contribution is the energy of mixing^33,34^

6 $$\psi_{m}=k_{b}T\left(n_{s}\ln\left(1-c_{p}\right)+N_{0}\ln c_{p}+\chi n_{s}c_{p}\right)$$

where *c*_*p*_ = 1/*J*_*s*_ is the volume fraction of the polymer in the hydrogel and χ is the dimensionless interaction parameter associated with the heat of mixing that accounts for the solvent-network interactions, and *p*_*s*_ is a Lagrange multiplier that enforces the incompressibility of the polymer and the water molecules and accounts for the osmotic pressure.

The true stress that develops in the swollen hydrogel is

7 $$\sigma_{s}=\frac{1}{J_{s}}\frac{\partial \psi_{s}}{\partial F_{s}}F_{s}^{T}=\frac{N_{0}}{J_{s}}\left\langle \sigma_{c}^{\left(s\right)}\right\rangle -p_{s}I$$

where

8 $$\sigma_{c}^{\left(s\right)}=f_{s}\frac{R_{s}^{2}}{r}F_{s}\mathrm{R̂}⊗F_{s}\mathrm{R̂}$$

is the stress on a chain.

The chemical potential of a water molecule in the network is

9 $$\mu =\frac{\partial \psi_{s}}{\partial m}=\mu_{0}+k_{b}T\left(\ln\left(1-c_{p}\right)+c_{p}+\chi c_{p}^{2}\right)+p_{s}v_{w}$$

where *v*_*w*_ is the volume of a water molecule and μ_0_ is the reference chemical potential of a water molecule in the aqueous bath.

### 2.2 The Collapsed State (*T* > *T*_VPTT_)

The local behavior of a chain in the collapsed phase is inherently different than that in the swollen state. Specifically, as shown in Figure 2, the application of a sufficiently large force leads to the pulling-out of chain segments from the collapsed globule, resulting in a random coil conformation.^24,40,41^ In addition, the collapsed PNIPAM network is hydrophobic and therefore swelling does not occur. In the following, the free energy-density of a collapsed gel that captures these two effects is developed.

Consider a dry PNIPAM network at a temperature *T* > *T*_VPTT_ comprising *N*_0_ chains per unit dry volume. An external force is applied and the material deforms. The material points in the undeformed and the deformed configurations are denoted by **x**_*g*_ and **y**_*g*_, respectively, such that the deformation gradient is **F**_*g*_ = ∂**y**_*g*_/∂**x**_*g*_. The network is assumed to be incompressible, and therefore det **F**_*g*_ = 1.

From a microscopic viewpoint, the chains are initially in a globule (collapsed) state in which intramolecular bonds inhibit chain mobility. On average, the chains have *n*_*g*_ repeat units with an effective contour length *L*_*g*_ such that the end-to-end distance is *R*_*g*_ = *L*_*g*_/*n*_*g*_. Once a sufficiently large force *f̃* is applied, the intramolecular bonds break (or, alternatively, chain segments are being pulled out) to release a “hidden-length”. As a result, chains gain additional mobility (or additional degrees of freedom) such that they comprise *n*_*s*_ > *n*_*g*_ repeat units with a contour length of *L*_*s*_ > *L*_*g*_. As a result, the end-to-end distance of the chain jumps from *r*_1_ to *r*_2_.

To capture this dependency, we define the ratio ρ_*m*_ = *r*/*L*_*s*_ as the ratio between the end-to-end distance and the contour length of the fully extended random coil (i.e., the chain in the swollen state), which enables one to write the ratio ρ_*g*_ = *r*/*L*_*g*_= ρ_*m*_ *L*_*s*_/*L*_*g*_. This allows to express the entropy and the local force on the chain (eq 2) below and above the critical force *f̃* in terms of ρ_*m*_. Specifically, the force on a chain is given via the piece-wise function^48,49^

10 $$f_{g}\left(\rho_{m}\right)=k_{b}T\{\begin{array}{ll} \frac{n_{g}}{L_{g}}\beta \left(\rho_{m}\frac{L_{s}}{L_{g}}\right) & 0\leq \rho_{m}<\rho_{1}\\ \mathrm{f̃} & \rho_{1}<\rho_{m}<\rho_{2}\\ \frac{n_{s}}{L_{s}}\beta \left(\rho_{m}\right) & \rho_{2}\leq \rho_{m}<1 \end{array}$$

where ρ_1_ = *r*_1_/*L*_*s*_ and ρ_2_ = *r*_2_/*L*_*s*_ are the ratios between the end-to-end distance and the extended contour length before and after the dissociation of the intramolecular bonds, respectively. The force-elongation response of a collapsed chain is depicted in Figure 3.

**Figure 3:**
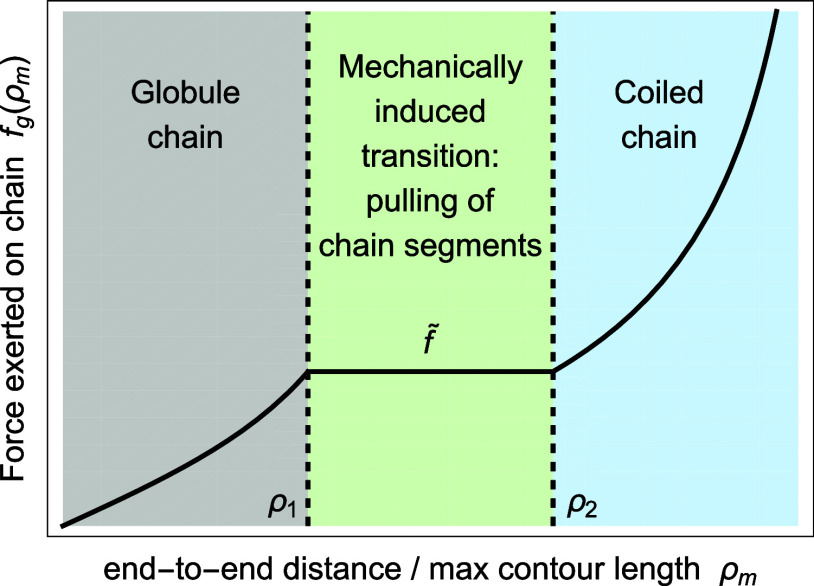
Force *f*_*g*_ exerted on a collapsed chain as a function of the ratio ρ_*m*_.

Consequently, the energy density of the collapsed PNIPAM network is

11 $$\psi_{g}=\psi_{n}\left(F_{g},R_{g},n_{s},n_{g},T\right)$$

and accordingly the stress is

12 $$\sigma_{g}=\frac{\partial \psi_{g}}{\partial F_{g}}F_{g}^{T}=N_{0}\left\langle \sigma_{c}^{\left(g\right)}\right\rangle -p_{g}I$$

where

13 $$\sigma_{c}^{\left(g\right)}=f_{g}\frac{R_{g}^{2}}{r}F_{g}\mathrm{R̂}⊗F_{g}\mathrm{R̂}$$

is the stress on a collapsed chain and *p*_*g*_ is a pressure-like term that enforces the incompressibility of the network.

## 3 The Mechanisms behind Phase Coexistence

To better understand the underlying mechanisms that enable phase coexistence in PNIPAM gels, we follow the work of Suzuki and Ishii^1^ and examine the uniaxial stretching of a collapsed rod around the VPTT. In the collapsed network, local extension of chains motivates the globule to coil transition by breaking intramolecular bonds and “pulling” chain segments out of the globule, thereby inducing elongated hydrophilic chains that attract water.^24,40,41^ Once a sufficient number of chains “open up” in the vicinity of one another, a local swollen domain emerges. This process is stochastic in nature, with a probability of extended chains that increases with the external force. Accordingly, swollen domains can form in a collapsed network, resulting in phase coexistence within the PNIPAM rod.

In this section, a model for the formation of phase coexistence is derived based on the above description.

### 3.1 Kinematics

Following the experiment of Suzuki and Ishii,^1^ consider a collapsed PNIPAM rod of length *B* and cross-sectional area *A*_*g*_. The network comprises *N*_0_ chains per unit dry volume (i.e., above the VPTT). We define a coordinate system {**x̂**, **ŷ**, **ẑ**} in which the rod is aligned along the **x̂**-direction. The rod stretches such that its overall length is *λB*, where λ is the axial stretch.

To capture phase coexistence, we define the critical stretches λ_1_ and λ_2_, such that for stretches λ < λ_1_ the rod is in a fully collapsed state and stretches λ > λ_2_ result in a fully swollen rod. In the range λ_1_ ≤ λ ≤ λ_2_, phase coexistence is observed. The origin of this behavior can be understood from the microstructure - for longitudinal stretches λ < λ_1_, the local forces are not sufficient to transition enough chains from a globule-like to a coiled conformation in order to trigger a phase transition. Once a stretch λ = λ_1_ is prescribed, many chains “open up” and become hydrophilic, leading to significant water uptake and the initiation of a swollen domain. Further increase in stretch (or external force) motivates the local transition of additional chains, thereby increasing the size of the swollen domains in the rod. At λ = λ_2_, all chains transitioned to coils and the rod is fully swollen. This process is depicted in Figure 4.

**Figure 4:**
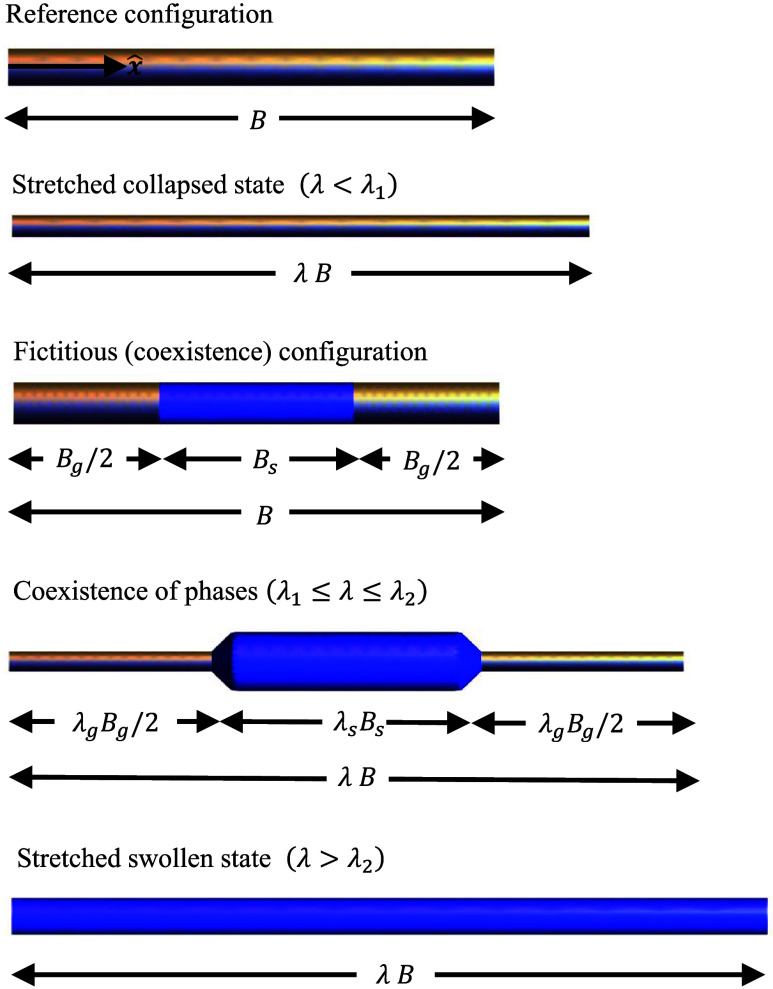
A schematic illustration of the stretching of a collapsed PNIPAM chain: a collapsed chain is stretched from the reference configuration by λ < λ_1_. Further extension (λ_1_ < λ < λ_2_) leads to the coexistence of phases - the rod is stretched from a fictitious to a deformed state comprising collapsed and swollen domains. At λ= λ_2_ the rod transitions completely to the swollen state, and further stretch (λ > λ_2_) leads to the elongation of a swollen rod.

For any stretch λ_1_ ≤ λ ≤ λ_2_, it is convenient to define a fictitious unloaded state in which the length of the collapsed and the swollen sections of the rod are *B*_*g*_= *B*_*g*_ (λ) and *B*_*s*_ = *B*_*s*_ (λ), respectively, such that *B* = *B*_*g*_ + *B*_*s*_. For completeness, it is stated that *B*_*g*_ (λ < λ_1_) = *B* and *B*_*s*_ (λ < λ_1_) = 0 (i.e., the rod is in a collapsed state for λ < λ_1_) and *B*_*g*_ (λ > λ_2_) = 0 and *B*_*s*_ (λ > λ_2_) = *B* (i.e., the rod is in a swollen state for λ > λ_2_). Next, the rod is stretched from this fictitious state to a length λ*B* = λ_*g*_ *B*_*g*_ + λ_*s*_ *B*_*s*_, where λ_*g*_ and λ_*s*_ are the local stretches of the collapsed and the swollen regions, respectively (see Figure 4).

Since the rod is uniaxially stretched, the deformation gradient of the collapsed and the swollen domains can be written as

14 $$F_{g}=\lambda_{g}\mathrm{x̂}⊗\mathrm{x̂}+\frac{1}{\sqrt{\lambda_{g}}}\left(ŷ⊗ŷ+ẑ⊗ẑ\right)$$

and

15 $$F_{s}=J_{s}^{1/3}\left(\lambda_{s}\mathrm{x̂}⊗\mathrm{x̂}+\frac{1}{\sqrt{\lambda_{s}}}\left(ŷ⊗ŷ+ẑ⊗ẑ\right)\right)$$

respectively.

### 3.2 Microstructural Conditions for Formation of Phases

To model the nucleation, or the initial formation of a swollen phase in a collapsed rod, recall that a force *f̃* must be applied locally on a chain in order to “pull-out” chain segments and break the globule structure.^24,40,41^ Following eq 10, this force corresponds to a ratio ρ_1_ ≤ ρ_*m*_ ≤ ρ_2_ between the end-to-end distance and *L*_*s*_, and therefore we expect the transition of a chain to occur in that range. To form a swollen domain in the network, a sufficient number of chains must “open up” to allow for water uptake.

To capture the stochastic nature of the globule-to-coil transition, an appropriate probability of transition 0 ≤ *P* ≤ 1 must be defined for which *P* (ρ_*m*_ < ρ_1_) = 0, corresponding to a collapsed chain, and *P* (ρ_*m*_ > ρ_2_) = 1, corresponding to a chain in an extended state. In this work, I employ a continuous truncated Gaussian-distribution based cumulative distribution function

16 $$P\left(\rho_{m}\right)=\frac{\mathrm{Erf}\left(\frac{\rho_{m}-\rho_{d}}{\sqrt{2}v}\right)+\mathrm{Erf}\left(\frac{\rho_{d}}{\sqrt{2}v}\right)}{\mathrm{Erf}\left(\frac{1-\rho_{d}}{\sqrt{2}v}\right)+\mathrm{Erf}\left(\frac{\rho_{d}}{\sqrt{2}v}\right)}$$

where 0 < ρ_*d*_ < 1 is a parameter that governs the ratio at which the chain transitions and *v* is the standard deviation. The probability is illustrated in Figure 5.

**Figure 5:**
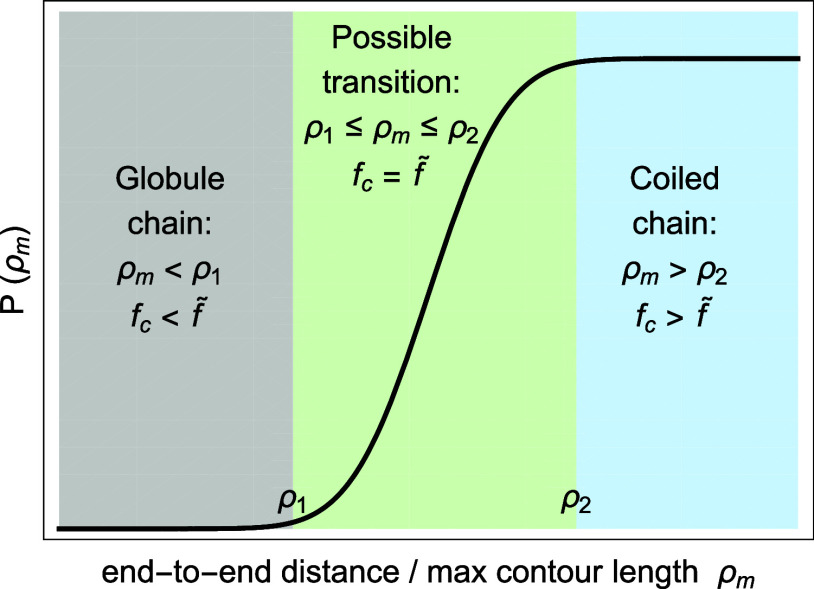
Illustration of the probability *P* (eq 16) as a function of the ratio ρ_*m*_ that a chain transitions from globule to coil.

The nucleation of a swollen domain requires the “pulling out” of chains in the network. One can compute the fraction of open chains as follows: first, we denote by ρ_*m*_^(*i*)^(θ^(*i*)^) chains with a referential end-to-end direction **R̂** ^(*i*)^ that form an angle θ^(*i*)^ ≤ θ < θ^(*i*)^ + Δ*θ* with the longitudinal direction **x̂**. The fraction of these chains in the network is proportional to the solid angle such that *p*_θ_(θ) = sin (θ) Δ*θ*/2, where 0 ≤ θ ≤ π. As the rod stretches, chains deform and one can write ρ_*m*_^(*i*)^ = ρ_*m*_^(*i*)^ (θ^(*i*)^, **F**). Note that chains with θ^(*i*)^ → 0 and θ^(*i*)^ → π extend, while chains along the transverse direction (i.e., θ^(*i*)^ ≈ π/2) shorten, with deformations that are proportional to the local deformation gradient. As expected, the stretching of the chains increases the likelihood of “opening up” to an extended conformation (see eq 16). The density of extended chains per unit dry volume along the *i*-th direction is given by *N*_*e*_^(*i*)^ = *P* (ρ_*m*_^(*i*)^) *p*_θ_ (θ) *N*_0_. Subsequently, one can compute the fraction of open chains in the network via

17 $$0\leq q=\frac{\sum_{i}N_{e}^{\left(i\right)}}{N_{0}}=\int_{0}^{\pi }P\left(\rho_{m}^{\left(i\right)}\right)p_{\theta }\left(\theta \right)d\theta \leq 1$$

where in passing the summation is converted to integration over all angles 0 ≤ θ ≤ π.

Before proceeding, it is worthwhile noting that chains along the plane transverse to the stretching direction shorten and therefore cannot naturally open up. The transition of these chains is enabled by the following mechanism: as chains get pulled out to an extended conformation, they attract water molecules from the environment, resulting in local swelling. The local presence of water molecules motivates all chains in the network to transition, ultimately leading to a macroscopically visible swollen phase.

To relate the local response to the macroscopic behavior, we conjecture that the fraction per length of the swollen domain is proportional to *q* such that *q* ≈ *B*_*s*_/*B*. By employing the relation λ*B* = λ_*g*_ *B*_*g*_ + λ_*s*_ *B*_*s*_, we relate the macroscopic deformation λ to the local deformations λ_*s*_and λ_*g*_ of the swollen and collapsed domains via

18 $$\lambda =\lambda_{g}\left(1-q\right)+\lambda_{s}q$$

Note that in the limit *q* = 0 the stretch λ = λ_*g*_, corresponding to a collapsed rod, whereas in the limit *q* = 1 the network is in its swollen configuration and the stretch λ = λ_*s*_.

### 3.3 Equilibrium Conditions

A rod in phase coexistence must satisfy mechanical and chemical equilibrium. Mechanical equilibrium requires that the stress in the two phases be divergence free, i.e., ∇ · **σ**_*s*_ = **0** and ∇ · **σ**_*g*_ = **0**. In addition, the longitudinal forces along the rod in the collapsed and the swollen domains must be equal, i.e., ∫**σ**_*g*_ **x̂** · **x̂**d*a*_*g*_ = ∫**σ**_*s*_ **x̂** · **x̂**d*a*_*s*_. The true stress in the swollen domains **σ**_*s*_ and the collapsed domains **σ**_*g*_ is given in eqs 7 and 12, respectively. To determine the deformed areas *a*_*g*_ and *a*_*s*_ in the collapsed and the swollen states, we assume that the two phases are incompressible and employ Nanson’s formula. In the case of uniaxial extension, the deformed areas are *a*_*g*_ = *A*_*g*_/λ_*g*_ and *a*_*s*_ = *A*_*s*_/λ_*s*_, where *A*_*g*_ and *A*_*s*_ are the areas of the fully collapsed and the fully swollen rods in the traction free configuration. These areas were experimentally measured by Suzuki and Ishii^1^ and Suzuki et al.^6^

Due to the hydrophilicity, the swollen domain must also satisfy chemical equilibrium, which pertains to the interactions between the solvent molecules and the gels. This is satisfied via μ = μ_0_ (see eq 9).

### 3.4 Integration from the Chain to the Network Level

To integrate from the chain to the network level, I employ the numerical microsphere technique.^47,50–52^ This method enables one to estimate the directional averaging of one-dimensional elements (the end-to-end vector directions **R̂**) over a unit sphere to obtain macroscopic quantities. Specifically, the stress that develops in a unit volume *dV*_*p*_ with *N*_0_ chains per unit volume can be determined via

19 $$\left\langle \sigma_{c}\right\rangle =\frac{1}{4\pi }\int_{A}\sigma_{c}dA\approx \sum_{i=1}^{m}\sigma_{c}^{\left(i\right)}w^{\left(i\right)}$$

where the summation is carried over *m* representative directions and the index *i* = 1, 2, ..., *m* refers to the *i*-th chain with an initial end-to-end direction **R̂** ^(*i*)^ and a non-negative weight *w* ^(*i*)^. Here, **σ**_*c*_ = **σ**_*c*_^(*s*)^ (eq 8) or **σ**_*c*_ = **σ**_*c*_^(*g*)^ (eq 13) to account for the stress of chains in the swollen or the collapsed domains, respectively.

It is emphasized that the weights are constrained such that ∑_*i*_ *w* ^(*i*)^ = 1. In addition, in isotropic networks such as the PNIPAM hydrogels, the representative directions must be chosen such that ∑_*i*_ **R̂** ^(*i*)^ *w* ^(*i*)^ = **0** and ∑_*i*_ **R̂** ^(*i*)^ ⊗ **R̂** ^(*i*)^ *w* ^(*i*)^ = 1/3**I**. Bažant and Oh^50^ showed that *m* = 42 specific representative directions and weights (given in Table 1 of that work) provide sufficient accuracy for isotropic materials, and this conclusion is used in this work.

**Table 1:** Summary of Model Parameters

notation	definition
*L*_*g*_/*L*_*s*_	contour length of collapsed/extended chain
*n*_*g*_/*n*_*s*_	number of repeat units in collapsed/extended chain
*f̃*	critical force to break intramolecular bonds in collapsed chain
ρ_*m*_	ratio between end-to-end distance *r* and *L*_*s*_
ρ_1_/ρ_2_	ratio between end-to-end distance and *L*_*s*_ before/after dissociation of intramolecular bonds
*J*_*s*_	volumetric deformation from dry to swollen networks
*N*_0_	chain-density per unit dry volume
*G* = *N*_0_ *k*_*b*_ *T*	shear modulus of dry network
λ_*g*_/λ_*s*_	uniaxial stretch in collapsed/swollen network
**σ**_*g*_/**σ**_*s*_	true stress in collapsed/swollen networks
*P*	probability of conformational transition of chain
*v*/ρ_*d*_	probability related parameters to govern transition
*q*	fraction of open chains in the network

## 4 Comparison to Experimental Findings

To validate the model, we summarize all of the model parameters in Table 1 for convenience and compare its predictions to the experimental findings reported in Suzuki and Ishii.^1^ To this end, we follow the experimental work of Liang and Nakajima^24^ and the corresponding model of Levin and Cohen^25^ and set the effective contour length of the extended and the collapsed chains *L*_*s*_ = 100 nm and *L*_*g*_ = 35 nm, respectively. To satisfy the continuity condition of the force on a collapsed chain (eq 10), we set the critical force *f̃* = 15 pN,^24^ ρ_1_ = 0.2, and ρ_2_ = 0.5 such that the number of Kuhn segments in the two states are *n*_*s*_ = 193 and *n*_*g*_ = 55.

From a macroscopic viewpoint, Suzuki and Ishii^1^ measured the diameters of the cylinder immediately before and after the VPTT (i.e., in the swollen and the collapsed phases) *d*_*s*_ = 110 μm and *d*_*g*_ = 58 μm, respectively. Accordingly, we can calculate the cross-sectional areas *A*_*s*_ and *A*_*g*_. In addition, the volume of a water molecule is *v*_*w*_ = 3 × 10^–29^ m^3^. To estimate the interaction parameter χ, we consider the traction free swelling of a PNIPAM rod from the collapsed to the swollen configuration. The volumetric deformation around the VPTT is assumed to be *J*_*s*_ = (*d*_*s*_/*d*_*g*_)^3^ ≈ 6.82, which enables one to determine χ ≈ 0.552 by setting **σ**_*s*_= **0** and μ = μ_0_ in eqs 7 and 9. This result corresponds to previous experimental findings on PNIPAM rods.^11,53^ It is emphasized that this is an approximation, since the classical swelling theory does not account for the curtailment of the chain from an extended to a globule state.

The stiffness of the collapsed gel *G* = *N*_0_ *k*_*b*_ *T*_VPTT_ = 22.7 kPa at the VPTT is fitted to the experimental findings. This value is within the range of measured stiffness values for PNIPAM, reported as ∼1–10 kPa and ∼10^2^–10^3^ kPa below and above the VPTT, respectively.^32,54,55^ The stiffness of the swollen network can be approximated via *G*/*J*_*s*_^1/3^.^33,44,47^

To capture the transition of local domains from collapsed to swollen (or swollen to collapsed), the standard deviation *v* = 5 × 10^–3^ and the parameter ρ_*d*_ = 0.115, pertaining to the transition probability in eq 16, are obtained from a fit to the experimental findings.

In the following, the two experiments performed by Suzuki and Ishii^1^ are considered and investigated.

### 4.1 Stretch Induced Phase Coexistence

We begin by comparing the model predictions to the stretch-induced phase coexistence demonstrated by Suzuki and Ishii.^1^ As illustrated in Figure 4, a collapsed PNIPAM rod was stretched at a temperature *T* ≈ *T*_VPTT_ (∼33.5 °C). Once a sufficient critical force was applied, a swollen phase began to appear. Further increase in force led to a higher ratio of swollen portion to total length until ultimately the rod transitions to a completely swollen continuum. It is again pointed out that since the rod is subjected to uniaxial tension, chains aligned along the longitudinal direction experience tension and are pulled-out first.

Figure 6a plots the ratio of the swollen portion as a function of the macroscopic stretch λ according to the experimental findings of Suzuki and Ishii^1^ (circle marks) and the model predictions (continuous curve). The model agrees with the experimental data. As shown in the work of Suzuki and Ishii,^1^ a macroscopic stretch λ ∼ 2.2 is required to initiate a swollen domain and at a stretch λ ∼ 3.3 the rod is fully swollen.

**Figure 6:**
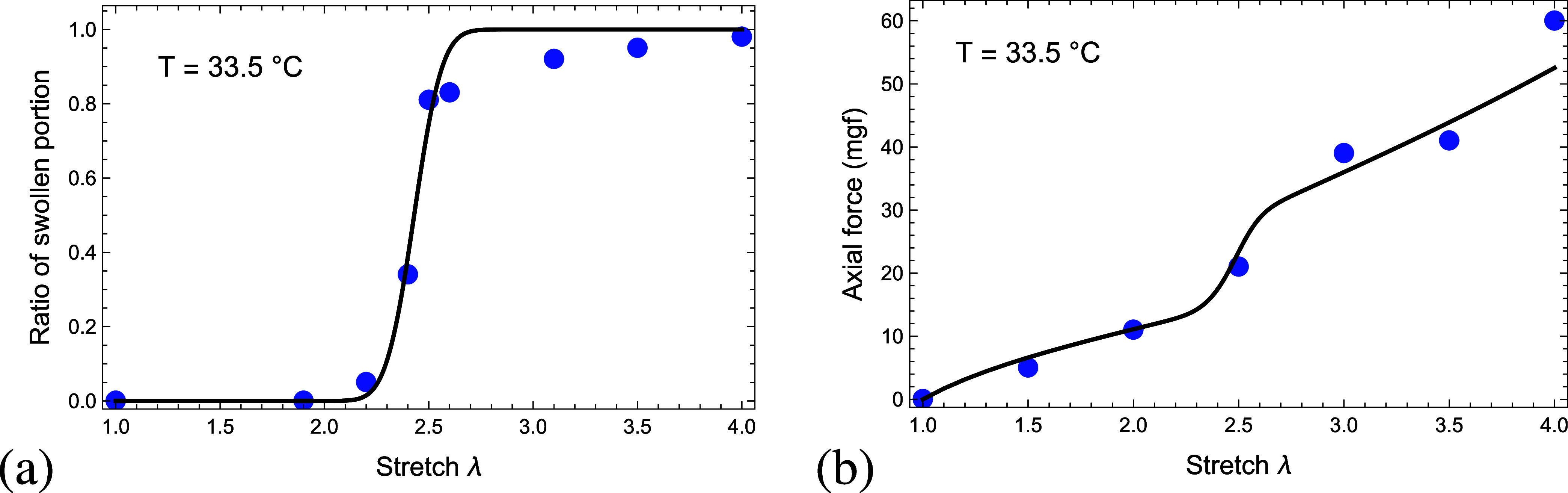
(a) The ratio of the swollen portion and (b) the axial force (in mgf) as a function of the macroscopic stretch λ. The curves correspond to the model predictions and the circular marks denote the experimental findings reported by (a) Suzuki and Ishii^1^ and (b) Suzuki et al.^6^

To further validate the model, we compute the axial force *f* = ∫**σ**_*s*_ **x̂** · **x̂**d*a*_*c*_ = ∫**σ**_*g*_ **x̂** · **x̂**d*a*_*s*_ as a function of the stretch λ. While Suzuki and Ishii^1^ did not report the axial force during this experiment, a previous work (Suzuki et al.,^6^ by the same author) measured the force on PNIPAM rods that were held fixed at a stretch λ and subjected to increasing temperature. As an estimate, one can take the forces measured at the temperature *T* = 33.5 °C under different stretch values and plot them (circle marks) against the model predictions (continuous curve), as shown in Figure 6b. Once again, the model agrees well with the experimental findings.

Further insights from the model can be gained by examining the local stretch in the collapsed (λ_*g*_) and the swollen (λ_*s*_) domains as a function of the macroscopic stretch λ, as depicted in Figure 7. Prior to the nucleation of the swollen phase, the stretch of the collapsed domain is the same as the macroscopic stretch, i.e., λ_*g*_ = λ. Once a swollen domain forms, the chains locally stretch due to swelling and the external force. We point out that Figure 7 only shows the stretch λ_*s*_ associated with a mechanical force, without considering the swelling-induced extension.

**Figure 7:**
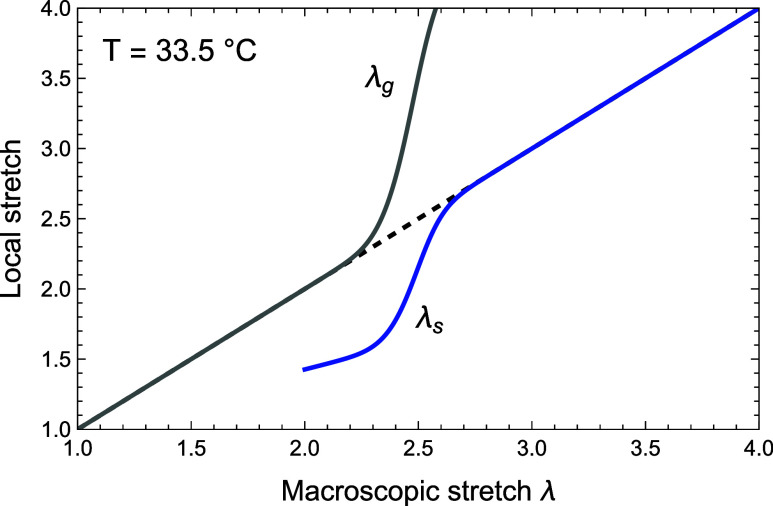
Local stretch in the collapsed (λ_*g*_) and the swollen (λ_*s*_) domains as a function of the macroscopic stretch λ (dashed line).

Interestingly, we find that while the force in the two domains is equal (to satisfy mechanical equilibrium), the stretch and the stress differ. To understand this, one must examine the cross-sectional area and the stiffness of the two domains. With respect to the former, the cross-sectional area of the swollen regions is larger than that of the collapsed ones (*A*_*s*_ > *A*_*g*_), as one would expect. Regarding the latter, the collapsed domains are dry (since the chains transition to their hydrophobic state) and are therefore stiff. On the other hand, the stiffness of the swollen domain is influenced by two factors - the softening due to water uptake and the stretch-induced stiffening of the chains, which must extend to accommodate water molecules.^33,44,47^ It is also underscored that the transition of the chain from a collapsed conformation to an extended coil is accompanied by an increase in the effective contour length, stemming from the dissociation of the intramolecular bonds. To ensure equilibrium during phase coexistence, the stress experienced by the collapsed domains is higher than the swollen regions, but due to its smaller cross-sectional area we find that the stretch λ_*g*_ is higher. Once all the chains transition to an extended state, the rod is in a fully swollen configuration and λ = λ_*s*_.

### 4.2 Relaxation-Induced Phase Coexistence

Suzuki et al.^6^ demonstrated that a swollen PNIPAM rod held at a fixed length and a critical temperature *T* ≈ *T*_VPTT_ (∼33.5 °C) occupies a configuration with phase coexistence as a function of time (see Figure 8). In this experiment, a swollen rod was held fixed at a constant length at *T* = 30 °C and heated to *T* = 33.5 °C. At this point, the thermal energy motivates the contraction of the chains to achieve a collapsed rod. In parallel, the external constraint gives rise to a tensile force that prevents the chains from contracting. As a result, a rod with phase coexistence develops. It is worth pointing out that further increase in temperature to *T* = 33.6 °C led to the disappearance of phase coexistence and the collapse of the tube.

**Figure 8:**
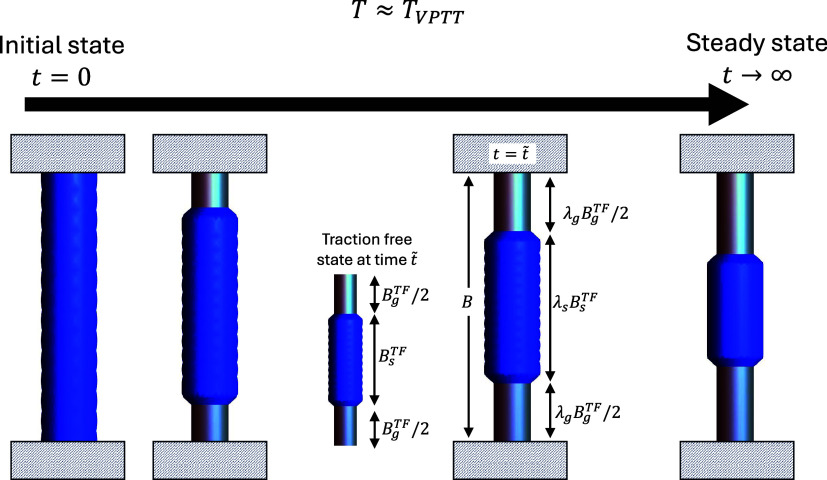
Time dependent coexistence of phases in a constrained PNIPAM rod at *T* ≈ *T*_VPTT_, as shown by Suzuki and Ishii.^1^

As opposed to the previous case, in which a collapsed PNIPAM rod was stretched to achieve phase coexistence, the rod was initially in the swollen phase with a fixed length *B* and collapsed domains form with time. At the beginning of the experiment, the chains in the swollen hydrogel have an average end-to-end distance $R=J_{s}^{\mathrm{TF}}/\sqrt{n_{s}}$ , where *J*_*s*_^*TF*^ ≈ 6.7 is the volumetric deformation determined from a traction free rod that is placed in an aqueous bath. Due to the thermal energy that is transferred to the PNIPAM rod from the environment, chains begin to collapse an in turn, tensile forces arise. To understand the time dependent microstructural evolution of the chains, it is important to note that chains which are aligned along the fiber direction experience tension due to the tensile force which prevents them from contracting. On the other hand, chains along the transverse plane shorten and are significantly more likely to transition to globule-like conformations.

Similar to the analysis of a stretch-induced phase coexistence, we determine the stretches λ_*s*_ and λ_*g*_ of the swollen and the collapsed domains, respectively, with respect to a fictitious traction free state. In this configuration, the lengths of the swollen and the collapsed domains are *B*_*s*_^*TF*^ and *B*_*g*_^*TF*^, respectively, as shown in Figure 8. In the beginning of the experiment (*t* = 0), *B*_*s*_^*TF*^ + *B*_*g*_^*TF*^ = *B* and the rod is traction free. At *t* > 0, the length of the traction free rod decreases *B*_*s*_^*TF*^ + *B*_*g*_^*TF*^ < *B* due to the outward flux of water molecules. To capture the time-dependent relaxation of the rod, we define the relaxation stretch from the referential to the traction free configuration at time *t*,

20 $$\lambda_{r}\left(t\right)=\left(1-\lambda_{r}^{\infty }\right)\;\exp\left(-\frac{t}{\tau }\right)+\lambda_{r}^{\infty }$$

where λ_*r*_ (*t* → ∞) = λ_*r*_^∞^ is the steady state traction free state and τ is a characteristic relaxation time. This constitutive relation can be viewed as rate of transition of the chains from the swollen to the collapsed phases. In the following, we fit λ_*r*_^∞^ = 0.825 and τ = 40 h. The stretch of the collapsed region is defined as the initial and the current relaxed lengths of the rod, i.e., λ_*s*_ = 1/λ_*r*_.

Figure 9a plots the ratio of the swollen portion as a function of time *t* (in hours). The circle marks denote the experimental data from Suzuki and Ishii^1^ and the continuous curve is the model prediction. The model is capable of capturing the experimental results. Experiments revealed that the swollen portion saturated after 200 h and remained in stable phase coexistence for several days, indicating a steady state.

**Figure 9:**
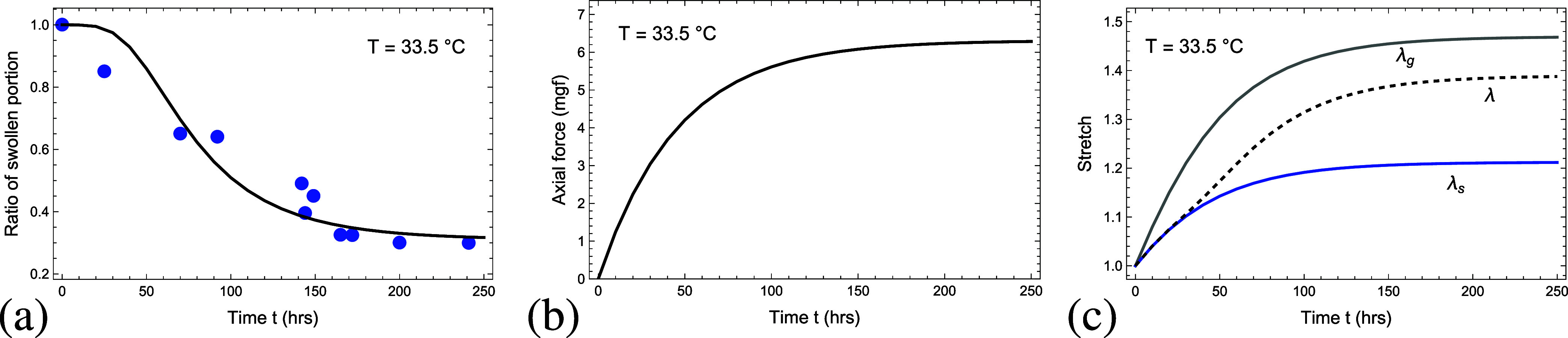
(a) The ratio of the swollen portion, (b) the axial force (in mgf), and (c) the total stretch λand the local stretch in the collapsed (λ_*g*_) and the swollen (λ_*s*_) domains as a function of time *t* (in hours). The curves correspond to the model predictions and the circular marks in (a) denote the experimental data from Suzuki and Ishii.^1^

While the tensile force that developed during the experiment was not reported, it was estimated with the model and is shown in Figure 9b. As expected, the force increases but remains small, corresponding to the measurements reported by Suzuki et al.^6^ (see the circular marks denoted by 1 in Figure 3a of that work). This prediction can be validated by comparing the maximum force at steady state *f*_*ss*_ ≈ 6.3 based on the model at *t* > 250 h to that which would be required to stretch a collapsed rod of length *B*_*c*_ to the original fixed length during the experiment *B*. Here, *B*_*c*_ is approximated via the configuration of the swollen gel in the dry state. Since the rod in the experiment of Suzuki and Ishii^1^ achieves stable phase coexistence, the axial force *f*_*ss*_ that develops is expected to be smaller than the force *f*_max_ which would be required to stretch a fully collapsed rod to the initial length. This calculation yields a maximum force *f*_max_ ≈ 10 mgf > *f*_*ss*_.

Lastly, Figure 9c depicts the total stretch λand the local stretch in the collapsed (λ_*g*_) and the swollen (λ_*s*_) domains as a function of time *t*. The collapsed domains form very quickly and are typically associated with a higher stretch. Once again, we find that the stretch λ_*g*_ is higher than λ_*s*_ for the same reasons as those described in reference to Figure 7.

### 4.3 A Note on the Influence of Geometry on the Coexistence of Phases

As mentioned by Suzuki and Ishii,^1^ geometry plays a significant role in the presence of phase coexistence and VPTT. To understand this, let us consider the initial (minimal) volume *v*_*n*_ that is associated with the onset of nucleation. Next, we examine the length to diameter ratio *d*/*L* of a dry cylindrical PNIPAM network subjected to uniaxial extension around the VPTT. In the limit *d*/*L* ≪ 1, extension leads to an initially stable nucleation of a swollen domain in the rod with a volume ∼*v*_*n*_. Further increase in tensile force enlarges the volume fraction of the swollen domain until ultimately the entire rod is in a swollen state. This case is the one considered in this work.

Alternatively, we can consider a dry PNIPAM disc with the dimensions *d*/*L* ≈ 1 such that the total volume of the network π*d* ^2^ *L*/4 < *v*_*n*_, i.e., the minimum stable nucleation volume is larger than the volume of the gel. Initial application of a uniaxial force leads to the extension of the collapsed disc. However, once a critical uniaxial force is applied, the globule-like chains are pulled out at once and an abrupt and complete collapsed-to-swollen transition occurs.

### 4.4 A Note on the Influence of Mechanical Constraints on the VPTT

The application of a mechanical force can be used to tune the VPTT. The work of Suzuki et al.^6^ demonstrated this with the following experiment: a swollen PNIPAM rod was stretched from its traction free state at *T* = 30 °C by a ratio λand held fixed, where the range of investigated stretches was λ = 1 – 6. Next, the PNIPAM was subjected to a slow temperature increase in increments of 0.05 °C, and the volume and the force that developed were measured.

It was shown that the VPTT increased from 33.45 °C at λ = 1 to slightly over 34.5 °C at λ = 6. In addition, the swollen-to-collapsed transition was accompanied by an increase in the force required to maintain the constant length for the stretches λ = 1–3.5, while a decrease in force was measured for λ > 3.5. Interestingly, the volumetric deformations also depend on the stretch and Suzuki et al.^6^ demonstrated that the stress increases upon the swollen-to-collapsed transition for all stretch values.

The observations in that work can be explained through the local microstructural evolution of the PNIPAM chains - at small fixed stretches λ the forces on the chains are small and the energetic cost of the coil-to-globule transition is low, but it is still higher than in a traction free network. As the prestretch λ increases, the chains experience higher forces which prevent the local transitions and raise the VPTT. While outside the scope of this contribution, these findings provide a pathway to tune the VPTT through mechanical constraints.

## 5 Conclusions

This contribution aims to explain the emergence of phase coexistence in PNIPAM rods around the VPTT in the presence of mechanical constraints, as demonstrated in the pioneering work of Suzuki and Ishii.^1^ That paper reported two interesting experiments that led to nontrivial behaviors: (1) the nucleation and time-dependent evolution of a collapsed phase in a fixed swollen PNIPAM rod and (2) the stretch-induced formation of swollen domains in a collapsed PNIPAM rod subjected to uniaxial extension.

To delineate the mechanisms that enable phase coexistence, I began by developing a local model for the PNIPAM chains. Chains below the VPTT are hydrophilic with a long contour length whereas chains above the VPTT are hydrophobic due to intramolecular interactions, which lead to a shorter contour length. As seen in various experiments, the application of a mechanical force to a globule-like chain leads to the dissociation of the intramolecular interactions, resulting in the “pulling-out” of chain segments that extend the contour length and motivate water uptake. The stress associated with swollen and collapsed networks was developed based on the local chain behavior.

Next, a method to describe the nucleation and evolution of a swollen phase in a collapsed PNIPAM network subjected to uniaxial extension was introduced. The local coil-to-globule transition is a stochastic event, and accordingly a probabilistic approach was employed to determine the state of a chain under a given mechanical force. The nucleation and evolution of swollen domains requires the transition of many local chains, and the likelihood of such an event was estimated from the local probabilities of the chains. It is emphasized that phase coexistence is maintained at equilibrium, and therefore the conditions for mechanical and chemical equilibrium were summarized.

To validate the proposed framework, I compared its predictions to the two experiments reported by Suzuki and Ishii.^1^ The model is capable of capturing the experimental findings and sheds light on the microstructural evolution and local stress that develops during the loading process.

The findings from this work show that mechanical constraints are the main stabilizing source that enables simultaneous presence of collapsed and swollen phases near the VPTT. Specifically, the deformation of collapsed PNIPAM hydrogels at a temperature *T* ∼ *T*_VPTT_ can lead to the pulling out of chains by breaking intramolecular bonds. Once extended, the PNIPAM chains are hydrophilic and attract water molecules in the environment, resulting in the nucleation of a swollen domain that grows in size as additional chains open up as a result of an increasing external force. It is important to point out that the stochasticity of force-induced conformational transitions plays a key role in deciding the position of the onset of nucleation and its growth.

It was demonstrated that a similar effect occurs in constrained swollen PNIPAM hydrogels that are heated to a temperature *T* ∼ *T*_VPTT_. In this case, the chains in the network tend toward a globule-like conformation. However, the external mechanical constraint hinders and delays the transition, resulting in a time-dependent response with a collapsed phase that increases in size until a steady state is reached.

Collectively, the findings from this work suggest a paradigm shift in the control over the VPTT from traditional chemical design toward mechanical force tuning. Specifically, as opposed to the classical approaches, in which the VPTT is programmed through changes to the chemical composition and network topology, this work sheds light and provides guidelines on how mechanical constraints can be employed to the same effect. Therefore, the proposed framework offers a pathway to improve the control and performance of PNIPAM-based systems in applications ranging from soft robotics to smart switches, where the ability to precisely manipulate phase behavior is critical.
